# Rap1a Regulates Cardiac Fibroblast Contraction of 3D Diabetic Collagen Matrices by Increased Activation of the AGE/RAGE Cascade

**DOI:** 10.3390/cells10061286

**Published:** 2021-05-22

**Authors:** Stephanie D. Burr, James A. Stewart

**Affiliations:** Department of BioMolecular Sciences, University of Mississippi School of Pharmacy, University, MS 38677, USA; jastewa7@olemiss.edu

**Keywords:** Rap1a, AGE/RAGE signaling, cardiac fibroblasts, collagen, 3D collagen matrix, diabetes

## Abstract

Cardiovascular disease is a common diabetic complication that can arise when cardiac fibroblasts transition into myofibroblasts. Myofibroblast transition can be induced by advanced glycated end products (AGEs) present in the extracellular matrix (ECM) activating RAGE (receptor for advanced glycated end products) to elicit intracellular signaling. The levels of AGEs are higher under diabetic conditions due to the hyperglycemic conditions present in diabetics. AGE/RAGE signaling has been shown to alter protein expression and ROS production in cardiac fibroblasts, resulting in changes in cellular function, such as migration and contraction. Recently, a small GTPase, Rap1a, has been identified to overlap the AGE/RAGE signaling cascade and mediate changes in protein expression. While Rap1a has been shown to impact AGE/RAGE-induced protein expression, there are currently no data examining the impact Rap1a has on AGE/RAGE-induced cardiac fibroblast function. Therefore, we aimed to determine the impact of Rap1a on AGE/RAGE-mediated cardiac fibroblast contraction, as well as the influence isolated diabetic ECM has on facilitating these effects. In order to address this idea, genetically different cardiac fibroblasts were embedded in 3D collagen matrices consisting of collagen isolated from either non-diabetic of diabetic mice. Fibroblasts were treated with EPAC and/or exogenous AGEs, which was followed by assessment of matrix contraction, protein expression (α-SMA, SOD-1, and SOD-2), and hydrogen peroxide production. The results showed Rap1a overlaps the AGE/RAGE cascade to increase the myofibroblast population and generation of ROS production. The increase in myofibroblasts and oxidative stress appeared to contribute to increased matrix contraction, which was further exacerbated by diabetic conditions. Based off these results, we determined that Rap1a was essential in mediating the response of cardiac fibroblasts to AGEs within diabetic collagen.

## 1. Introduction

Heart failure is a common diabetic complication arising from the transition of cardiac fibroblasts into myofibroblasts within the myocardium [1,2]. The myofibroblast population in cardiac tissue has been shown to be higher in diabetics and contribute to left ventricle dysfunction and heart failure [3,4,5]. The presence of myofibroblasts is denoted by increased alpha smooth muscle actin (α-SMA) expression and characterized by increased extracellular matrix (ECM) remodeling, oxidative stress, cell-mediated matrix contraction, and migration [4,6,7,8]. The myofibroblast transition can be influenced by advanced glycated end products (AGEs) embedded within the ECM. AGEs can bind to their receptor (RAGE, receptor for advanced glycated end products) to elicit the activation of a multifaceted signaling cascade that contributes to downstream events, such as increased oxidative stress, ECM remodeling, and myofibroblast differentiation [9,10].

Activation of the AGE/RAGE signaling cascade is able to alter protein expression resulting in changes to cellular function. For instance, AGE activation of RAGE led to increased protein kinase C (PKC) and extracellular signal related-kinase 1/2 (ERK1/2) activation to trigger the assembly and activity of NADPH oxidase, resulting in increased ROS production [5,11,12,13,14]. Increased PKC, ERK1/2, and ROS production can also promote ECM remodeling, denoted by increased collagen synthesis and MMP activity [5,15,16,17,18]. Increased superoxides generation AGE/RAGE signaling can be reduced by increased expression of SODs converting superoxides into hydrogen peroxide [19,20,21]. In addition to ROS production, the AGE/RAGE signaling cascade can elicit myofibroblast differentiation, marked by increased α-SMA expression, which has been shown to increase collagen matrix contraction [22,23,24]. In vivo studies have found activation of AGE/RAGE signaling was correlated with increased fibrosis associated with increased myofibroblast activity [9,25,26,27]. Examinations of in vitro α-SMA expression levels have shown conflicting reports in which AGE/RAGE cascade activation can either increase or decrease these protein levels within cells [28,29,30]. This discrepancy with α-SMA protein expression as a result of RAGE signaling could possibly be due to various cellular and molecular components, such as ROS levels and ECM components affecting the cellular response to increased signaling. Therefore, it is important when utilizing an in vitro model to assess the impact of the ECM microenvironment on changes in cellular function.

The ECM can act as a physical scaffold and a reservoir for signaling proteins, which both can alter cellular behavior [31,32,33]. Our studies using extracted diabetic ECM allows for in vitro models to recapitulate the in vivo microenvironment. Studies have shown that ECM components, such as collagen and fibronectin, can be used to generate a 2D or 3D environment; however, the 3D environment better represents the in vivo environment [31]. For example, pulmonary fibroblasts cultured on 3D type 1 collagen matrix displayed higher levels of α-SMA protein expression, leading to increased cellular matrix contraction [24]. Similarly, it has been demonstrated that cells in a 3D environment exhibited changes in morphology, proliferation, and migration compared to cells in a 2D environment [31,34,35,36]. Additionally, the presence of molecular components within the ECM can induce changes in cellular behavior and protein expression [37]. The presence of ECM collagen can lead to integrin α2β1-mediated fibroblast adhesion to induce cellular contraction of a 3D matrix, which was prevented by blocking integrin α2β1 [38,39]. Furthermore, AGEs, a molecular ECM component bound to collagen isolated from diabetic mice, can cause an increase in cardiac fibroblast contraction of a 3D collagen matrix [7]. These studies highlighted how the ECM and its molecular components can impact cellular behavior.

AGE/RAGE cascade downstream events can be modified by a small GTPase, Rap1a. Repressor/activator protein 1 (Rap1a) has been shown to be involved in connecting extracellular stimuli to intracellular responses by eliciting changes in effector proteins [40,41,42,43]. In addition, Rap1a has been demonstrated to play a role in cellular signaling within the developing and adult myocardium [40,41]. Recently, our lab has demonstrated that Rap1a overlaps the AGE/RAGE cascade in cardiac fibroblasts and contributes to myofibroblast differentiation by maintaining α-SMA expression, as well as regulating oxidative stress by modulating changes in NF-κB and SOD-1 expression [30]. In addition, Rap1a contributed to AGE/RAGE-mediated decreases in cardiac fibroblasts migration, possibly by regulating PKC-ζ and ERK1/2 activity [8]. Similar studies have shown that Rap1a can operate through PKC and ERK1/2 to increase migration and proliferation of vascular smooth muscle cells [44]. Rap1a may also influence oxidative stress due to its ability to bind to the subunit p22^PHOX^, a component of the NADPH oxidase system, and its interactions with PKC-ζ have also been shown to phosphorylate NAPDH subunit p47^PHOX^, promoting formation of the NADPH oxidase system [45,46,47,48]. Several signaling proteins influenced by Rap1a are also linked to AGE/RAGE signaling [17,44,49]. While many studies of Rap1a examined the influence on expression of signaling proteins, very few have examined the impact of Rap1a on in vitro cellular function, specifically contraction.

Therefore, we propose to examine the impact of Rap1a on AGE/RAGE-mediated cardiac fibroblast contraction, as well as the influence of isolated diabetic ECM on facilitating these effects. This study aims to evaluate three specific ideas: (1) assess the role Rap1a plays in AGE/RAGE-mediated cardiac fibroblast contraction; (2) determine the effect of oxidative stress on mediating fibroblast contraction; and (3) examine how the source of ECM collagen impacts cardiac fibroblast contractile response. We hypothesized that Rap1a contributes to an AGE/RAGE-mediated increase in oxidative stress and myofibroblast transition, which affects cardiac fibroblast contraction under diabetic conditions. To accomplish this goal, we isolated cardiac fibroblasts from genetically different mice and embedded them into a 3D collagen matrix composed of extracted collagen from either non-diabetic or diabetic mouse tails. Fibroblasts were then treated with exogenous AGEs and/or EPAC to stimulate RAGE and Rap1a activity, respectively, for a 7-day time period, which was akin to chronic exposure of cells to external stimuli. Assessments of matrix contraction, expression of proteins associated with AGE/RAGE signaling, α-SMA, SOD-1, and SOD-2, and hydrogen peroxide levels were conducted. These results determined that, in a 3D collagen environment, Rap1a overlapped the AGE/RAGE cascade to increase myofibroblast differentiation and oxidative stress, resulting in increased matrix contraction, which was further exacerbated by diabetic conditions.

## 2. Methods and Materials

### 2.1. Animal Models

Male *Lepr^db^* (db/db model; BKS Cg-*DOCK7^m^* +/+ *Lepr^db^*, Jackson Labs; JAX# 00642) at 16 weeks of age were used as a type 2 diabetic animal model. The db/db model contains a nonfunctional leptin receptor as a result of a point mutation. The mutation led to the development of insulin resistance and obesity, which resulted in hyperglycemia by 8 weeks of age and overt diabetes by 12 weeks of age. Lean controls consisted of heterozygous male mice (non-diabetic). RAGE knockout mice were generated by using the Cre-Lox recombination methodology. Briefly, RAGE exons 2–7 were flanked by two loxP sites in the same orientation and then exposed to Cre recombinase (via breeding with Cre delete mice), which led to the deletion of RAGE exons 2–7 [50,51,52,53]. This deletion targets the cytoplasmic tail of RAGE which results in a global loss of RAGE signaling. In addition, a reversely orientated transcriptional EGFP reporter gene was inserted into intron 7, this acted as a confirmation for the deletion of RAGE exons 2–7. Assessment for the expression of EGFP in RAGE knockout mice acted as a control to confirm knockout of RAGE. Experimental evaluation for the loss of RAGE exons 2–7 and expression of EGFP was documented in Burr et al. 2020 (https://doi.org/10.6084/m9.figshare.11299253, accessed on 23 March 2020) [7]. Generation of RKO non-diabetic (non-diabetic RKO) and diabetic (diabetic RKO) mice were generated by crossbreeding RKO mice to heterozygous mice [7,50,51]. Male Rap1a knockout mice (Rap1a KO) were established by insertion of a neomycin resistant gene after exon 4 of RAP1A in the opposite (3′–5′) orientation. The resistance gene was inserted via a 0.95 kb *Pyull-Ndel* fragment targeting vector to disrupt Rap1a mRNA expression [54].

### 2.2. Animal Care

Standard environmental conditions (12 h/12 h light/dark cycle) were used for housing animals. Animals were maintained on commercial mouse chow and tap water ad libitum. This study followed the principles of the National Institutes of Health “Guide for the Care and Use of Laboratory,” [55]. The University of Mississippi Institutional Animal Care and Use Committee approved the animal protocol utilized with this study (protocol #20-017). At 16 weeks of age, which was considered the experimental endpoint, euthanasia was performed by anesthesia by CO_2_ inhalation, followed by a second mechanism for euthanasia, cervical dislocation.

### 2.3. Cardiac Fibroblast Isolation and Culture

Hearts were removed from chest cavity with aortas and great vessels dissected away, leaving the ventricles for cell isolation. The ventricle tissue was weighed (Table 1) and then cut into approximately 5 mm pieces and placed in a collagenase-trypsin enzymatic solution (100 U/mL collagenase II, Worthington Biochemical; 0.1% Trypsin, Gibco) under sterile conditions [3]. Continuous mixing of cardiac tissue and enzymatic solution generated a single cell suspension after 1–1.5 h. The cell suspension was centrifuged to pellet the cells which was followed by resuspension in high glucose DMEM (high glucose media; Dulbecco’s Modified Eagles Medium DMEM) containing 4.5 g/L glucose, sodium pyruvate, L-glutamine, and supplemented with 14.2 mM NaHCO3, 14.9 mM HEPES, 1% L-glutamine, 0.02% Primocin™ (Thermo Fisher), and 15% heat-inactivated FBS (fetal bovine serum) for 24 h in an incubator (5% CO_2_; 37 °C). After 24 h, the unattached fibroblasts were removed by washing cells three times with appropriate media (non-diabetic and Rap1a fibroblasts: low glucose or euglycemic media; 1 g glucose/L and diabetic fibroblasts: high glucose or hyperglycemic media; 4.5 g glucose/L) and then incubated at 37 °C. One cell isolation contained 2–3 hearts and equaled one sample (*n* = 1). Cardiac fibroblasts were cultured until passage 1, which typically occurred 1–2 weeks after isolation. For clarity and simplicity, cardiac fibroblasts isolated from a specific mouse line were referred to by their descriptor (i.e., fibroblasts isolated from diabetic mice were referred to as diabetic fibroblasts).

### 2.4. Collagen Extraction

Tails isolated from non-diabetic and diabetic 16-week-old male mice were collected at the time of euthanasia and stored at 20 °C until used. The tails were washed in 70% ethanol and then collagen was extracted from mice tails by removing the tail skin to expose the 4 major tendons [56]. The tendons were removed and placed in a conical flask containing 150 mL acetic acid (1:1000 dilution in dH_2_O). The tendons were then cut into 5 mm pieces and mixed for 3 days at 4 °C. Hereinafter all steps were conducted in sterile conditions. After 72 h, the solution containing tendon and acetic acid was centrifuged at 3000× *g* for 30 min at 4 °C. Supernatant was removed and centrifuged at 3000× *g* for 30 min at 4 °C. The collagen supernatant was removed and stored at 4 °C. A Sircol^TM^ Soluble Collagen Assay Kit (Biocolor Ltd., Carrickfergu, UK) was used, as per manufacturer directions, to determine collagen concentration.

### 2.5. 3-Dimensional Collagen Matrix

Isolated collagen was combined with sterile 0.2 M HEPEs and 10× minimum essential media (MEM, Thermo Fisher) on ice in an 8:1:1 ratio to generate 3D collagen discs [57]. This was performed with collagen isolated from non-diabetic and diabetic mouse tails. The collagen solution was degassed on ice for 10 min to remove air bubbles. After degassing, equal amounts of collagen mixture and fibroblast cell suspension were combined. A total of 1 mL of the collagen-fibroblast solution was added per well in 24-well plate. The collagen-fibroblast solution was polymerized for 1 h at 37 °C. After polymerization, the collagen-fibroblast disc was dislodged from the sides of the well using a sterile P200 pipette tip and media plus pharmacological modifiers were added. Pharmacological modifiers consisted of EPAC (100 μm; 8-(4-Chlorophenylthio)-2′-O-methyladenosine 3′,5′-cyclic monophosphate monosodium hydrate, a Rap1 activator) and exogenous AGEs (0.5 mg/mL). The collagen-fibroblast collagen discs were incubated for 7 days at 37 °C. The media was not changed, and no additional drug dosages were added during this incubation period. Again, for simplicity, we chose to use the descriptive terms non-diabetic and diabetic with the collagen matrix to define the source of the matrices, using this nomenclature does not imply they are non-diabetic or diabetic.

### 2.6. Matrix Contraction Analysis

An iBRIGHT imaging system (Thermo Fisher Scientific, Waltham, MA, USA) was utilized to capture images of the collagen matrices and a ruler was used for scale reference. Specific imaging specifications were set at: Zoom: 1.2 and Focus: 2.16. Measurement of the area of the ring structure was done using Image J software (NIH) and the ruler was used to set the scale for each image. The area of the ring structure was set as the space from the center point of the contractile ring to the inner perimeter of the ring structure [6,58,59]. The area of the contractile ring was normalized to a set “control”. This “control” was designated as the average contractile ring area displayed by untreated, non-diabetic fibroblasts embedded in non-diabetic collagen (*n* = 10) [7]. This group was set as the “control” due to it best embodying how cardiac fibroblasts would act normally when exposed to a 3D collagen matrix. The values for the area of the ring structure for untreated, non-diabetic fibroblasts embedded in non-diabetic matrix for each sample were averaged, which generated one “control” value. The average “control” area value was used to normalize the results obtained from other genotypes and treatment groups.

### 2.7. Western Blot Analysis

Modified Hunter’s buffer (MHB; 75 mM NaCl, 0.5 mM orthovanadate, 5 mM tris pH 7.4, 0.5 mM ECTA, 0.25% NP-40, 0.5 mM EGTA, 1% Triton X-100, and freshly added Halt Protease Inhibitor Cocktail (100X; Thermo Fisher)) was utilized to isolate protein from fibroblasts embedded in collagen matrices. Matrices and MHB were incubated for 10 min on ice, followed by sonication. Protein samples were centrifuged for 15 min at 32,000× *g* at 4 °C, followed by another round of sonication and centrifugation using the same conditions as before. Multiple rounds of sonication were required to ensure the matrix was disrupted, which yields higher protein concentrations. A bichinchoninic acid assay (BCA: Pierce Biotechnology) determined the protein concentration, following the manufacturer’s instructions for each sample. Western blot analysis used equal protein concentrations (20 μg) per sample. Antibodies used were α-smooth muscle actin (α-SMA, 43 kDa; 1:400; Sigma Aldrich 2547), superoxide dismutase-1 (SOD-1, 23 kDa; 1:400; Santa Cruz Biotechnology sc-101523), and superoxide dismutase-2 (SOD-2, 25 kDa; 1:400; Santa Cruz Biotechnology sc-133134). Brilliant Blue Coomassie Stain was used to label total protein and was used as a loading control [60,61]. An iBRIGHT imaging system captured images of Western blots and Image J was used to conduct analysis. All original Western blot images are presented at the following location, 10.6084/m9.figshare.14150306 (date accessed 2 March 2021).

### 2.8. Immunofluorescence

Cardiac fibroblasts embedded in non-diabetic and diabetic collagen matrices were fixed using histology grade 4% paraformaldehyde (Fisher Scientific) for 10 min at room temperature. Matrices were washed 3 times with 1X PBS and then incubated in blocking solution (2% BSA [Fisher Scientific], 3% donkey serum [Fisher Scientific], 0.01% Triton X-100 in 1X PBS) overnight at 4 °C. Primary antibody directly conjugated phalloidin-cy3 (1:20; Thermo Fisher), was added to matrices and incubated overnight at 4 °C. Unbound primary antibody was removed by 3, washed for 5-min with 1X PBS, followed by an incubation for 1.5 h at room temperature with DAPI (1:1000; Fisher Scientific). A Nikon Eclipse Ti2 microscope with pco.edge camera (PCO) was used to image matrices.

### 2.9. Hydrogen Peroxide Assay

Hydrogen peroxide levels were assessed using protein lysates. The OxiSelect hydrogen peroxide/peroxidase assay kit (BioLabs, STA-344) was used following manufactures instructions. In brief, protein samples were diluted (1:2) in assay buffer solution and incubated for 30 min at room temperature with hydrogen peroxide working solution. The colorimetric assay was assessed with spectrometer at 540 nm. The concentration of hydrogen peroxide was determined for each sample using a standard curve.

### 2.10. Matrix Transmission Assay

Isolated non-diabetic and diabetic collagens were incubated with 10X MEM and 0.2M HEPEs on ice in an 8:1:1 ratio. Air bubbles were removed by degassing the collagen solution for 10 min on ice. Degassed collagen solution was mixed with equal volume of media and then 200 μL of collagen/media solution was plated into each well of a 96-well plate. Matrices were either untreated or treated with 4 μL of exogenous AGEs (0.334 mg/mL). A concentration of 0.334 mg/mL was selected due to primary studies indicating higher concentrations of AGE do not elicit a greater response (data not shown). Matrix/media solution incubated at 37 °C for 1 h, followed by spectrometric analysis at 400 nm. The optical density value was used to calculate percent transmission (equation: 10^−OD^*100). Two different collagen isolations for non-diabetic collagen and one collagen isolation for diabetic collagen were used with this assay and included 22 replicates. 

### 2.11. Matrix Compaction Assay

The compaction assay used within this study was adapted from Howard et al. (1996) using non-diabetic and diabetic collagen matrices [59]. The collagen matrices were prepared as previously described. A total 1 mL of collagen/media solution was added to 1.5 mL microcentrifuge tube and incubated for 1 h at 37 °C to allow for gel polymerization to occur. After gel polymerization, the initial height of the collagen matrix was marked on the microcentrifuge tube. Collagen matrices were centrifuged at 5000× *g* for 5 min at room temperature. After centrifugation, the height of the compacted matrix was indicated on the microcentrifuge tube. However, due to the collagen matrix compacting at an angle, the highest and lowest heights of the compacted matrix were marked. An average was taken of the final high and low heights, which generated a value that more accurately represented the compacted height of the matrix.

### 2.12. Statistical Analysis

**Figure 1:**
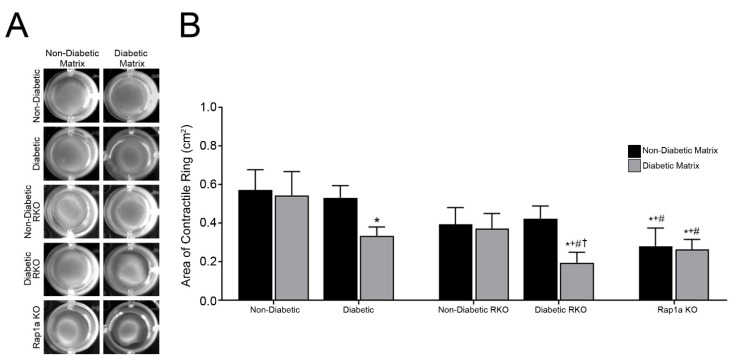
The presence of RAGE, Rap1a, and isolated ECM altered the area of contractile ring in cardiac fibroblasts. Cardiac fibroblasts were isolated from non-diabetic, diabetic, non-diabetic RKO, diabetic RKO, and Rap1a KO hearts and embedded in either non-diabetic or diabetic collagen matrix for 7 days. (**A**) Representative images of cardiac fibroblasts embedded in non-diabetic and diabetic collagen. (**B**) Depiction of the area (mean ± SEM) of the contractile ring with *n* = 5–10. Significance was determined by a two-way ANOVA followed by a Fisher’s Least Significant Difference post hoc. Symbols above bars indicate significance between two designated groups (*p* < 0.05; * vs. non-diabetic fibroblasts in non-diabetic matrix, + vs. non-diabetic fibroblast in diabetic matrix, # vs. diabetic fibroblasts in non-diabetic matrix, and † vs. diabetic RKO fibroblasts in non-diabetic matrix). Black bars represent cardiac fibroblasts embedded in non-diabetic collagen and gray bars indicate cells embedded in diabetic collagen.

**Figure 2:**
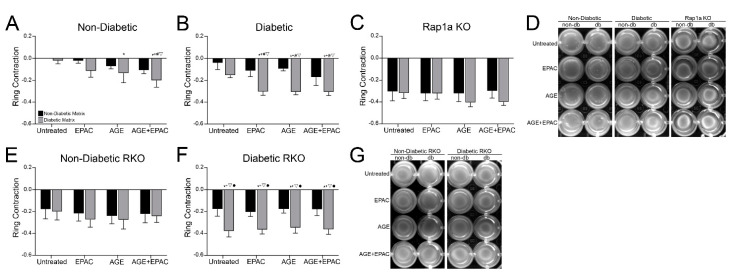
Cardiac fibroblast matrix contraction was altered by differing levels of AGE/RAGE signaling and diabetic matrix components. Hearts from (**A**) non-diabetic, (**B**) diabetic, (**C**) Rap1a KO, (**E**) non-diabetic RKO, and (**f**) diabetic RKO mice were used to isolate cardiac fibroblasts. Fibroblasts were embedded in non-diabetic (non-db; black bar) and diabetic (db; gray bar) collagen matrix and treated with EPAC (100 µM), exogenous AGEs (0.5 mg/mL), or EPAC+exogenous AGEs (100 µM and 0.5 mg/mL, respectively). After 7 days, images of matrices were captured and the area of the contractile ring was assessed. Data depicted in graphs are differences in contractile rings between treatment groups and the control group. The control group was set as untreated non-diabetic fibroblasts embedded in non-diabetic matrix. The control group was set to 0, with a positive value indicating a larger ring area (less matrix contraction), while a negative value denotes a smaller ring area (more matrix contraction) compared to control group. (**D**,**F**,**H**) Representative matrix images for each genotype and treatment. Data are mean ± SEM, *n* = 5–10, and statistical analysis consisted of a two-way ANOVA followed by a Fisher’s Least Significant Difference post hoc. Symbols above specific bars denote significant difference compared to a specific treatment group (*p* < 0.05; * vs. untreated in non-db matrix, + vs. untreated in db matrix, # vs. EPAC in non-db matrix, ▽ vs. AGE in non-db matrix, and • vs. AGE+EPAC in non-db matrix).

**Figure 3:**
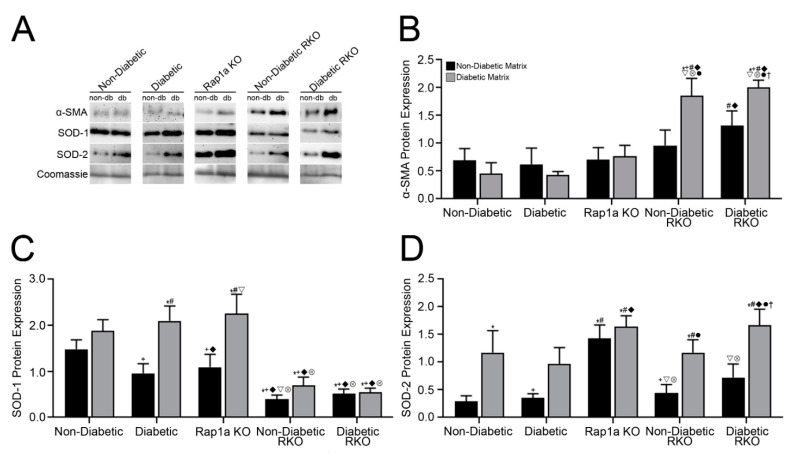
Isolated ECM and genotypic variation induced significant changes in expression of proteins associated with RAGE signaling. Cardiac fibroblasts isolated from non-diabetic, diabetic, Rap1a KO, non-diabetic RKO, and diabetic RKO were embedded in either non-diabetic (black bar) or diabetic (grey) collagen and cultured for 7 days. (**A**–**D**) Isolated protein was used for Western blot analysis to determine changes in protein expression. Western blot analysis for (**B**) α-SMA (42 kDa), (**C**) SOD-1 (23 kDa), and (**D**) SOD-2 (25 kDa) was normalized to total protein (Brilliant Blue Coomassie Stain), and graphs depict mean ± SEM (*n* = 5–10). Representative Western blot images shown in A are not displayed as a continuous blot due to limitations regarding number of samples and running lanes, but samples of the same genotype were run on the same blot. Statistical analysis consisted of a two-way ANOVA followed by a Fisher’s Least Significant Difference post hoc. Symbols above each bar indicate significance between a specific group (*p* < 0.05; * vs. non-db cells in non-db, + non-db cells in db, # db cells in non-db, ◆ vs. db cells in db, ▽ vs. Rap1a KO cells in non-db, ⊗ Rap1a KO cells in db, • non-db RKO cells in non-db, and † vs. db RKO in non-db).

**Figure 4:**
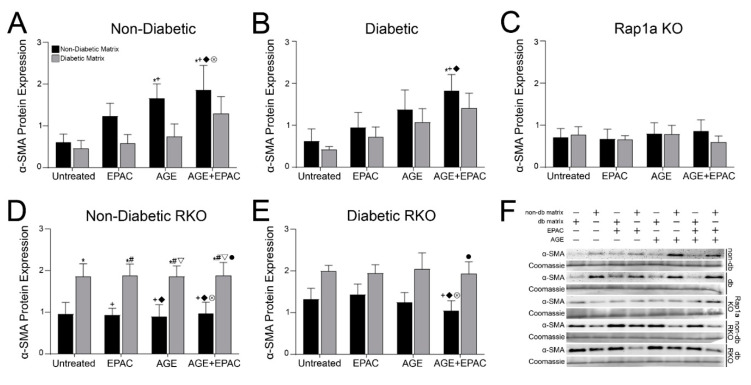
Isolated collagen and Rap1a mediated AGE/RAGE-induced increase of α-SMA expression in cardiac fibroblasts wildtype for RAGE. Cardiac fibroblasts were isolated from (**A**) non-diabetic, (**B**) diabetic, (**C**) Rap1a KO (**D**) non-diabetic RKO, and (**E**) diabetic RKO mouse hearts. Cells were embedded into non-diabetic (black bar) or diabetic (grey bar) collagen matrices for 7 days and then used to isolate total protein. Quantification of Western blot analysis for α-SMA (42 kDa) was assessed and normalized to total protein (Brilliant Blue Coomassie Stain) in untreated, EPAC (100 µM), exogenous AGEs (0.05 mg/mL) and EPAC+exogenous AGEs (100 µM and 0.05 mg/mL, respectively) cardiac fibroblasts. (**F**) Representative Western blot images for each treatment and genotype (non-db = non-diabetic matrix and db matrix = diabetic matrix). Data are means ± SEM with *n* = 5–10. Statistical analysis consisted of a two-way ANOVA followed by a Fisher’s Least Significant Difference post hoc. Symbols above each bar indicate significance between a specific group (*p* < 0.05; * vs. untreated in non-db, + vs. untreated in db, # vs. EPAC in non-db, ◆ vs. EPAC in db, ▽ vs. AGE in non-db, ⊗ vs. AGE in db, and • vs. AGE+EPAC in non-db). Untreated treatment groups presented were initially presented in Figure 3B.

**Figure 5:**
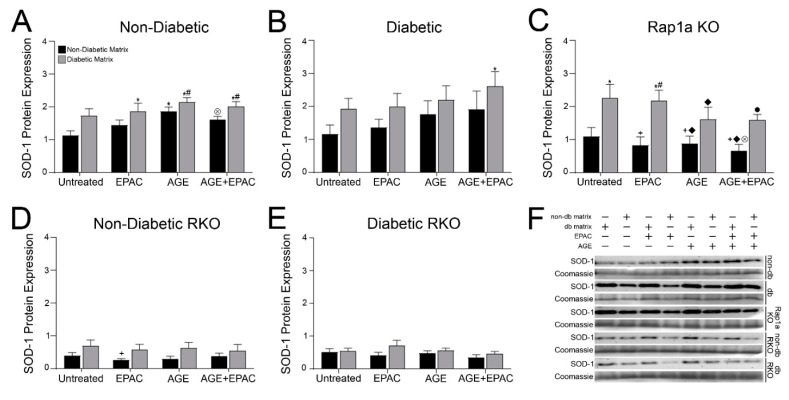
AGEs within diabetic collagen induced changes in SOD-1 expression utilizing both the AGE/RAGE signaling cascade and Rap1a. Cardiac fibroblasts from (**A**) non-diabetic, (**B**) diabetic, (**C**) Rap1a KO, (**D**) non-diabetic RKO, and (**E**) diabetic RKO were embedded in non-diabetic (black bars) and diabetic (grey bars) matrices. Protein expression was assessed by Western blot analysis for SOD-1 (23 kDa) and normalized to total protein (Brilliant Blue Coomassie Stain) in untreated, EPAC (100 µM), exogenous AGEs (0.05 mg/mL), and EPAC+exogenous AGEs (100 µM and 0.05 mg/mL, respectively) cardiac fibroblasts. (**F**) Representative Western blot images for SOD-1 protein expression and non-db = non-diabetic matrix and db matrix = diabetic matrix. Data are means ± SEM with *n* = 5–10. Statistical analysis consisted of a two-way ANOVA followed by a Fisher’s Least Significant Difference post hoc. Symbols above each bar indicate significance between a specific group (*p* < 0.05; * vs. untreated in non-db, + vs. untreated in db, # vs. EPAC in non-db, ◆ vs. EPAC in db, ⊗ vs. AGE in db, and • vs. AGE+EPAC in non-db). Untreated treatment groups presented were initially presented in Figure 3C.

**Figure 6:**
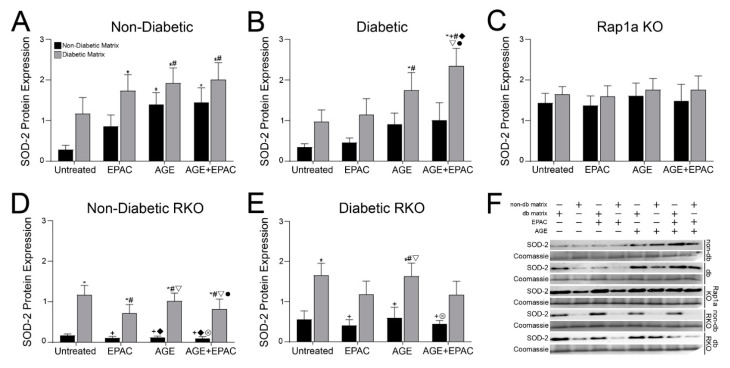
Diabetic matrix and AGE/RAGE signaling impacted expression of SOD-2 in cardiac fibroblasts. Isolated cardiac fibroblasts from (**A**) non-diabetic, (**B**) diabetic, (**C**) Rap1a KO, (**D**) non-diabetic RKO, and (**E**) diabetic RKO mouse hearts and embedded in non-diabetic (black bars) and diabetic (grey bars) matrices. Quantification of Western blot analysis for SOD-2 (25 kDa) was conducted and normalized to total protein (Brilliant Blue Coomassie Stain) in EPAC (100 µM), exogenous AGEs (0.05 mg/mL) and EPAC+exogenous AGEs (100 µM and 0.05 mg/mL, respectively) cardiac fibroblasts. (**F**) Representative Western blot images for SOD-2 protein expression in cardiac fibroblasts and non-db = non-diabetic matrix and db matrix = diabetic matrix. Data are means ± SEM with *n* = 5–10. Statistical analysis consisted of a two-way ANOVA followed by a Fisher’s Least Significant Difference post hoc. Symbols above each bar indicate significance between a specific group (*p* < 0.05; * vs. untreated in non-db, + vs. untreated in db, # vs. EPAC in non-db, ◆ vs. EPAC in db, ▽ vs. AGE in non-db, ⊗ vs. AGE in db, and • vs. AGE+EPAC in non-db). Untreated treatment groups presented were initially presented in Figure 3D.

**Figure 7:**
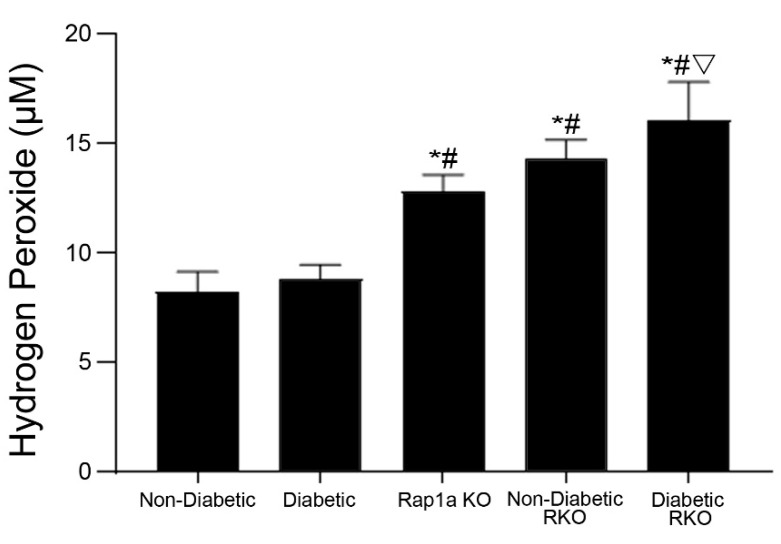
Rap1a KO and RKO cardiac fibroblasts embedded in non-diabetic collagen exhibited significantly higher concentrations of hydrogen peroxide. Cardiac fibroblasts isolated from non-diabetic, diabetic, Rap1a KO, non-diabetic RKO, and diabetic RKO mouse hearts were embedded in non-diabetic collagen for 7 days. Protein lysates were isolated and examined for concentration of hydrogen peroxide. Mean ± SEM were graphed with *n* = 5–10 and statistical analysis consisted of a one-way ANOVA, followed by a Fisher’s Least Significant Difference post hoc. Symbols above each bar indicate significance between a specific group (*p* < 0.05; * vs. non-diabetic in non-db, # vs. diabetic in non-db, ▽ vs. Rap1a KO in non-db).

**Figure 8:**
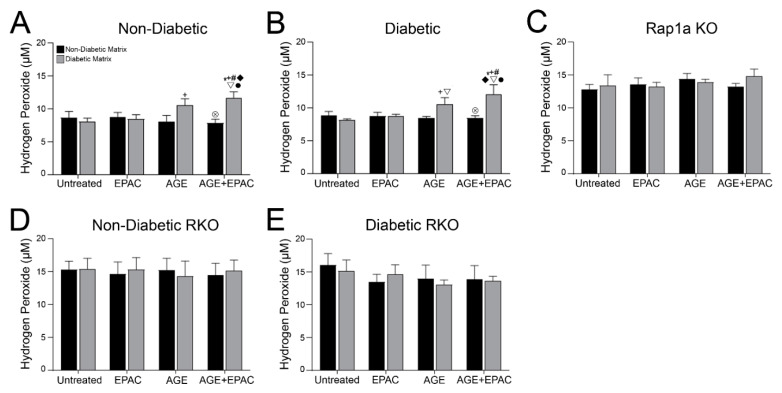
AGE/RAGE signaling and Rap1a expression impacted hydrogen peroxide levels in cardiac fibroblasts. Cardiac fibroblasts isolated from (**A**) non-diabetic, (**B**) diabetic, (**C**) Rap1a KO, (**D**) non-diabetic RKO, and (**E**) diabetic RKO mouse hearts. Fibroblasts were embedded in non-diabetic (black bars) and diabetic (grey bars) matrices for 7 days and treated with EPAC (100 µM), exogenous AGEs (0.05 mg/mL) and EPAC+exogenous AGEs (100 µM and 0.05 mg/mL, respectively). Protein lysates were collected and assessed for hydrogen peroxide concentration. Mean ± SEM were graphed with *n* = 5–10 and statistical analysis consisted of a two-way ANOVA, followed by a Fisher’s Least Significant Difference post hoc. Symbols above each bar indicate significance between a specific group (*p* < 0.05; * vs. untreated in non-db, + vs. untreated in db, # vs. EPAC in non-db, ◆ vs. EPAC in db, ▽ vs. AGE in non-db, ⊗ vs. AGE in db, and • vs. AGE+EPAC in non-db). Untreated treatment groups embedded in non-diabetic collagen were initially presented in Figure 7.

Statistical analysis was conducted using Graph Prism software, version 9.0.0. Both one-way and two-way ANOVAs were used to determine significance between variables. A one-way ANOVA was used for Figure 7 and Supplementary Figure S2, while a two-way ANOVA was used for Figures 1–6 and 8. A two-way ANOVA provided three *p* values which distinguished whether variables had an independent effect or whether an interaction between the variables had a significant impact. The variables for Figures 1 and 3 were collagen matrix and genotype, and the variables for Figures 2, 4–6 and 8 were collagen matrix and treatment. Following the ANOVA, a Fisher’s Least Significant Difference post hoc was conducted to determine significant differences between specific groups/treatments. All values presented within the graphs represent mean ± SEM. Symbols on graphs that denote significance are similar across graphs that depict similar data. Graphs that compared between different genotypes (Figures 1, 3 and 7) used the following symbols to denote which groups were compared: *, vs. non-diabetic cells in non-diabetic matrix; +, non-diabetic cells in diabetic matrix; #, diabetic cells in non-diabetic matrix; ◆, vs. diabetic cells in diabetic matrix; ▽, vs. Rap1a KO cells in non-diabetic matrix; ⊗, Rap1a KO cells in diabetic matrix; •, non-diabetic RKO cells in non-diabetic matrix; and †, vs. diabetic RKO in non-diabetic matrix. Graphs that compared between different treatment groups (Figures 2, 4–6 and 8) used the following symbols to denote which groups were compared: *, vs. untreated in non-diabetic matrix; +, vs. untreated in diabetic matrix; #, vs. EPAC in non-diabetic matrix; ◆, vs. EPAC in diabetic matrix; ▽, vs. AGE in non-diabetic matrix; ⊗, vs. AGE in diabetic matrix; •, vs. AGE+EPAC in non-diabetic matrix; and †, vs. AGE+EPAC in diabetic matrix.

## 3. Results

### 3.1. The Presence of 3D Non-Diabetic Collagen Matrix-Induced Changes in Cell Morphology and Rearrangment of Cardiac Fibroblasts Independent of RAGE

Cardiac fibroblasts embedded in a 3D collagen matrix exhibited more cellular projections, which were narrow and elongated in shape, compared to cells in a 2D system (Supplementary Figure S1). In addition, there appeared to be more concentrated points of F-actin in cells embedded in a 3D environment. The physical characteristics of non-diabetic and diabetic 3D collagen matrices differed, where the percent of transmission of diabetic collagen (untreated and AGE treated) was significantly less than non-diabetic collagen (Supplementary Figure S2A; one-way ANOVA *p* < 0.0001). Furthermore, examination of matrix compaction showed diabetic collagen, with or without AGEs, compacted significantly less compared to non-diabetic matrices (Supplementary Figure S2B; one-way ANOVA *p* < 0.0001). While physical characteristics differed between non-diabetic and diabetic collagen, cardiac fibroblasts embedded in both sources of collagen rearranged to form a circular ring structure (referred to as a contractile ring), which surrounded a cell-based network (Supplementary Figure S3). The presence of a 3D collagen network caused changes in morphology and cellular organization.

### 3.2. The Presence of RAGE, Rap1a, and Isolated ECM Altered the Area of the Contractile Ring in Cardiac Fibroblasts

The formation of the contractile ring allowed the cardiac fibroblasts to modify their environment though changes in cellular contractile functions. In order to assess the change in cellular contractile function, the area of the contractile ring was measured for each sample (Figure 1A). The results determined that both types of matrix (non-diabetic and diabetic) and genetic make-up of cardiac fibroblast had a significant impact on the area of the contractile ring (Figure 1B; two-way ANOVA, Interaction *p* = 0.4226, Matrix *p* = 0.0463, and Genotype *p* = 0.0049). Non-diabetic fibroblasts embedded in both non-diabetic and diabetic matrices exhibited the largest contractile ring area, which was significantly greater than diabetic and diabetic RKO cells embedded in a diabetic matrix. There were no significant differences in areas of contractile rings between non-diabetic and non-diabetic RKO fibroblasts in either matrix. Diabetic RKO cells had a smaller contractile ring area than both diabetic and diabetic RKO cells embedded in non-diabetic matrix. Lastly, Rap1a KO cells displayed a smaller contractile area than non-diabetic cells in both non-diabetic and diabetic matrices, as well as diabetic cells in non-diabetic matrix. The results suggest that the presence of AGE/RAGE signaling and Rap1a expression affected the area of the contractile ring, and therefore cellular contraction.

### 3.3. Cardiac Fibroblast Matrix Contraction Was Impacted by AGE/RAGE Signaling and Diabetic Matrix Components

In order to determine the impact of AGE/RAGE signaling and Rap1a on mediating changes to cardiac fibroblast contractile function, wildtype cells for RAGE and Rap1a were embedded in non-diabetic and diabetic matrices and treated with pharmacological modifiers to enhance AGE/RAGE signaling and Rap1a activity (Figure 2A,B). The area of the contractile ring of non-diabetic fibroblasts was significantly affected by both increased AGE/RAGE and Rap1a signaling as well as the diabetic matrix (Figure 2A; two-way ANOVA, Interaction *p* = 0.8383, Matrix *p* = 0.0380, and Treatment *p* = 0.0198). Non-diabetic cells within a diabetic matrix exhibited more matrix contraction with increased AGE/RAGE signaling. AGE treated non-diabetic cells contracted the diabetic significantly more than non-diabetic cells in a non-diabetic matrix. Combined AGE+EPAC treatment resulted in significantly more matrix contraction in non-diabetic cells in the diabetic matrix compared to non-diabetic fibroblasts embedded in non-diabetic matrices. Similarly, the contractile function of diabetic cardiac fibroblasts was significantly impacted by AGE/RAGE and Rap1a signaling and the diabetic matrix (Figure 2B; two-way ANOVA, Interaction *p* = 0.7460, Matrix *p* < 0.0001, and Treatment *p* = 0.0317). Diabetic fibroblasts embedded in a diabetic matrix and treated with EPAC, AGE, and AGE+EPAC exhibited significantly greater matrix contraction compared to diabetic cells embedded in non-diabetic matrices, except for AGE+EPAC diabetic cells in non-diabetic matrix. The data suggest that higher levels of exogenous and endogenous AGEs, in combination with increased Rap1a activity, led to greater fibroblast contraction.

Cardiac fibroblasts that lacked RAGE or Rap1a exhibited different patterns in matrix contraction when treated with varying pharmacological modifiers and matrices compared to wildtype cells for RAGE (Figure 2C–G). The area of the contractile ring of Rap1a KO fibroblasts was not affected by AGE/RAGE signaling or matrix components (Figure 2C; two-way ANOVA, Interaction *p* = 0.8068, Matrix *p* = 0.2572, and Treatment *p* = 0.8216). Non-diabetic RKO cardiac fibroblasts responded in a similar manner to Rap1a KO cells in which matrix contraction was not altered by changes in AGE/RAGE signaling or matrix components (Figure 2E; two-way ANOVA, Interaction *p* = 0.9946, Matrix *p* = 0.5412, and Treatment *p* = 0.8185). However, diabetic RKO fibroblasts contractile ring area was affected by the presence of diabetic matrix components (Figure 2E; two-way ANOVA, Interaction *p* = 0.9799, Matrix *p* < 0.0001, and Treatment *p* = 0.9766). Diabetic cells contracted the diabetic matrix significantly more than diabetic RKO cells embedded in a non-diabetic matrix, independently of different treatment groups. These results indicate that AGE/RAGE signaling and Rap1a were involved with regulating the amount of fibroblast contraction. 

### 3.4. Isolated ECM and Genotype Variations Induced Significant Changes in Expression of Proteins Associated with RAGE Signaling

Proteins from untreated cardiac fibroblasts of studied genotypes embedded in non-diabetic and diabetic matrices were isolated and assessed for expression of proteins typically associated with AGE/RAGE signaling (Figure 3). Expression of α-SMA was only significantly impacted by the genotype of the cells (Figure 3B; two-way ANOVA, Interaction *p* = 0.0682, Matrix *p* = 0.1171, and Genotype *p* < 0.0001). Both non-diabetic and diabetic RKO cardiac fibroblasts displayed significantly more α-SMA expression as compared to the other groups of fibroblasts (non-diabetic, diabetic, and Rap1a KO), regardless of matrix. Diabetic RKO cells in non-diabetic collagen also displayed higher levels of α-SMA, but was only significant when compared to non-diabetic and diabetic cells embedded in a diabetic matrix.

In contrast to the pattern noted for α-SMA, expression of SOD-1 was higher in cardiac fibroblasts wildtype for RAGE and Rap1a. This finding was further exacerbated when cells were embedded in a diabetic matrix (Figure 3C; two-way ANOVA, Interaction *p* = 0.0424, Matrix *p* < 0.0001, and Treatment *p* < 0.0001). Non-diabetic, diabetic, and Rap1a KO cardiac fibroblast embedded in diabetic collagen matrix exhibited significantly more SOD-1 compared to non-diabetic and diabetic RKO fibroblasts embedded in either non-diabetic or diabetic matrices. Furthermore, diabetic and Rap1a KO cells both displayed significant differences in SOD-1 expression when embedded in a diabetic matrix compared to a non-diabetic matrix.

The expression pattern of SOD-2 was different than was noted with α-SMA and SOD-1 expression, where both genotypic variation and matrix components significantly impacted the expression of SOD-2 in cardiac fibroblasts (Figure 3D; two-way ANOVA, Interaction *p* = 0.4810, Matrix *p* < 0.0001, and Genotype *p* = 0.0006). In general, cardiac fibroblasts embedded in diabetic matrices displayed more SOD-2 compared to cells embedded in non-diabetic matrices. However, the differences were only significant with non-diabetic, non-diabetic RKO, and diabetic RKO cardiac fibroblasts. Rap1a KO cell in non-diabetic and diabetic matrices displayed the highest levels of SOD-2, which was significantly greater than the other cells embedded in non-diabetic matrices. Similarly, diabetic RKO fibroblasts embedded in a diabetic matrix exhibited significantly higher levels of SOD-2 compared to cells within non-diabetic matrices. The data indicated that the presence of RAGE, Rap1a, and diabetic collagen can impact the expression of AGE/RAGE-associated proteins.

### 3.5. Isolated Collagen and Rap1a-Mediated AGE/RAGE-Induced Increased α-SMA Expression in Cardiac Fibroblasts Wildtype for RAGE

Cardiac fibroblasts embedded in 3D collagen matrices were assessed for changes in α-SMA expression, a protein commonly associated with downstream AGE/RAGE signaling and fibroblast activation. Expression of α-SMA in non-diabetic fibroblasts embedded in collagen matrices was significantly impacted by AGE/RAGE signaling and matrix components (Figure 4A; two-way ANOVA, Interaction *p* = 0.7825, Matrix *p* = 0.0320, and Treatment *p* = 0.0405). Non-diabetic fibroblasts in non-diabetic matrix exhibited more α-SMA expression compared to cells in diabetic matrices. α-SMA expression was significantly higher in AGE- and AGE+EPAC-treated non-diabetic cells in non-diabetic matrices compared to untreated non-diabetic cells embedded in non-diabetic and diabetic matrices. Diabetic cardiac fibroblast α-SMA expression was only significantly affected by AGE/RAGE signaling (Figure 4B; two-way ANOVA, Interaction *p* = 0.9879, Matrix *p* = 0.2442, and Treatment *p* = 0.0174). Diabetic cells treated with AGE+EPAC embedded in a non-diabetic matrix exhibited significantly more α-SMA compared to untreated diabetic fibroblasts and EPAC treated diabetic cells in a diabetic matrix.

Cardiac fibroblasts that lacked Rap1a or RAGE exhibited changes in α-SMA expression that differed from wildtype cells for RAGE. Rap1a KO cells did not display any significant changes in α-SMA expression when exposed to different pharmacological modifiers and matrices (Figure 4C: two-way ANOVA, Interaction *p* = 0.8790, Matrix *p* = 0.7038, and Treatment *p* = 0.9493). Expression of α-SMA in non-diabetic RKO fibroblasts was significantly impacted by the type of matrix (Figure 4D; two-way ANOVA, Interaction *p* = 0.9994, Matrix *p* < 0.0001, and Treatment *p* = 0.9983). Non-diabetic RKO fibroblasts embedded in diabetic matrix exhibited significantly higher levels of α-SMA expression compared to cells embedded in non-diabetic matrices. Similarly, expression of α-SMA in diabetic RKO cells was significantly affected by the source of matrix (Figure 4E; two-way ANOVA, Interaction *p* = 0.8886, Matrix *p* = 0.0004, and Treatment *p* = 0.8650). Expression of α-SMA was significantly lower in diabetic RKO fibroblasts embedded in a non-diabetic matrix compared to diabetic RKO cells embedded in diabetic matrices. The results suggest that the increase in α-SMA expression was mediated by increased AGE/RAGE signaling and Rap1a activity.

### 3.6. AGEs in Diabetic Collagen Induced Changes in SOD-1 Expression Utilizing Both the AGE/RAGE Signaling Cascade and Rap1a

Cardiac fibroblasts wildtype for RAGE exhibited changes to SOD-1 expression induced by exposure to varying pharmacological modifiers and matrices (Figure 5). Non-diabetic fibroblast exhibited significant changes in SOD-1 expression due to both AGE/RAGE signaling and matrix (Figure 5A; two-way ANOVA; Interaction *p* = 0.8444, Matrix *p* = 0.0010, and Treatment *p* = 0.0113). SOD-1 expression was significantly higher in non-diabetic cells in non-diabetic matrix treated with AGEs compared to untreated non-diabetic fibroblasts in non-diabetic matrix. Furthermore, AGE and AGE+EPAC treated non-diabetic cells in diabetic matrix displayed significantly higher levels of SOD-1 compared to EPAC treated non-diabetic cells in non-diabetic matrix. SOD-1 expression in diabetic fibroblasts was only impacted by matrix components (Figure 5B; two-way ANOVA, Interaction *p* = 0.9825, Matrix *p* = 0.0388, and Treatment *p* = 0.3154). AGE+EPAC-treated diabetic cells in a diabetic matrix had significantly more SOD-1 expression compared to untreated diabetic cells in a non-diabetic matrix. Expression of SOD-1 in Rap1a KO cells was significantly altered by the source of collagen matrix (Figure 5C; two-way ANOVA, Interaction *p* = 0.7340, Matrix *p* < 0.0001, and Treatment *p* = 0.2899). Overall, it appeared that Rap1a KO cells in diabetic matrices displayed more SOD-1 compared to Rap1a KO cells in non-diabetic matrices. Specifically, untreated and EPAC treated Rap1a KO cells in diabetic matrix exhibited significantly higher levels of SOD-1 than all other Rap1a KO cells embedded in non-diabetic matrices. Treatment with AGEs appeared to reduce SOD-1 expression in Rap1a KO cells in diabetic matrices to levels that were no longer significantly different compared to cells in non-diabetic matrices.

Cardiac fibroblasts that lacked RAGE had a different response in SOD-1 expression as compared to cells with RAGE. Expression of SOD-1 was only significantly impacted by the source of collagen in non-diabetic RKO fibroblasts (Figure 5D; two-way ANOVA, Interaction *p* = 0.9372, Matrix *p* = 0.0069, and Treatment *p* = 0.8352), while diabetic RKO cells’ regulation of SOD-1 expression was not significantly affected by either AGE/RAGE signaling or matrix components (Figure 5E; two-way ANOVA, Interaction *p* = 0.5783, Matrix *p* = 0.0717, and Treatment *p* = 0.4421). These data indicated that AGE/RAGE signaling with Rap1a expression resulted in higher levels of SOD-1 expression in cardiac fibroblasts.

### 3.7. Diabetic Matrix and AGE/RAGE Signaling Impacted Expression of SOD-2 in Cardiac Fibroblasts

Regulation of SOD-2 expression in cardiac fibroblasts was changed by the presence of AGE/RAGE signaling, as well as the source of collagen (Figure 6). Non-diabetic fibroblasts SOD-2 expression was significantly increased by AGE/RAGE signaling and matrix components (Figure 6A; two-way ANOVA, Interaction *p* = 0.9188, Matrix *p* = 0.0052, and Treatment *p* = 0.0296). SOD-2 expression was greater in non-diabetic cells embedded in diabetic matrices. Treatment with AGE and AGE+EPAC of non-diabetic fibroblasts embedded in either non-diabetic or diabetic matrices exhibited significantly higher levels of SOD-2 compared to untreated non-diabetic cells in a non-diabetic matrix. Similarly, expression of SOD-2 in diabetic cardiac fibroblasts was significantly increased by both AGE/RAGE signaling and the source of collagen (Figure 6B; two-way ANOVA, Interaction *p* = 0.7126, Matrix *p* = 0.0005, and Treatment *p* = 0.0118). In general, diabetic fibroblasts embedded in diabetic matrix display more SOD-2 compared to diabetic cells in a non-diabetic matrix. However, only diabetic fibroblasts in a diabetic matrix treated with AGE+EPAC had significantly more SOD-2 expression compared to all other treatment groups, except AGE-treated diabetic cells in a diabetic matrix. The results indicated that increased AGE/RAGE signaling and Rap1a activity led to an increase in SOD-2 expression in cardiac fibroblasts.

The source of collagen matrices had varying impacts on RKO and Rap1a KO cardiac fibroblasts’ SOD-2 expression. Rap1a KO cells exhibited a different pattern of SOD-2 expression between non-diabetic and diabetic matrices as compared to fibroblasts with Rap1a. Expression of SOD-2 in Rap1a KO cells was not changed by either AGE/RAGE signaling or matrix components (Figure 6C; two-way ANOVA, Interaction *p* = 0.9971, Matrix *p* = 0.2948, and Treatment *p* = 0.8976). In contract, non-diabetic RKO fibroblast SOD-2 expression was significantly higher in diabetic matrices (Figure 6D; two-way ANOVA, Interaction *p* = 0.7157, Matrix *p* < 0.0001, and Treatment *p* = 0.4953). Non-diabetic cells embedded in a diabetic matrix had significantly more SOD-2 expression compared to treatment groups in which non-diabetic cells were embedded in a non-diabetic matrix. Diabetic RKO showed a similar trend in which SOD-2 expression was also significantly higher in diabetic matrices (Figure 6E; two-way ANOVA, Interaction *p* = 0.8919, Matrix *p* < 0.0001, and Treatment *p* = 0.4815). However, only untreated and AGE treated diabetic RKO cells had significantly greater SOD-2 expression compared to diabetic RKO cells embedded in non-diabetic matrices. Treatment with EPAC resulted in a loss of significant differences between diabetic RKO cells in a diabetic matrix and diabetic RKO cells in non-diabetic matrices. These results indicate that both Rap1a and the source of collagen can impact SOD-2 expression in cardiac fibroblasts.

### 3.8. Cardiac Fibroblasts with Knocked out RAGE Exhibited Significantly Higher Concentration of Hydrogen Peroxide

The level of oxidative stress within cardiac fibroblasts was assessed by measuring the concentration of hydrogen peroxide (H_2_O_2_), which was measured in untreated cardiac fibroblasts embedded in a non-diabetic matrix (Figure 7). Both RKO and Rap1a KO cardiac fibroblasts exhibited significantly more H_2_O_2_ (Figure 7; one-way ANOVA *p* < 0.0001). Non-diabetic and diabetic fibroblasts had significantly less H_2_O_2_ compared to Rap1a KO, non-diabetic RKO, and diabetic RKO cells. In addition, diabetic RKO cells had significantly higher levels of H_2_O_2_ compared to Rap1a KO cells.

### 3.9. AGE/RAGE Signaling and Rap1a Expression Altered Hydrogen Peroxide Levels in Cardiac Fibroblasts

Another assessment of downstream AGE/RAGE signaling was determined by measuring H_2_O_2_ concentrations in cardiac fibroblasts embedded in non-diabetic and diabetic matrices and treated with different pharmacological modifiers (Figure 8). Non-diabetic cardiac fibroblasts’ H_2_O_2_ production was significantly impacted by the source of the collagen matrix (Figure 8A; two-way ANOVA, Interaction *p* = 0.0226, Matrix *p* = 0.0246, and Treatment *p* = 0.3195). Non-diabetic fibroblasts embedded in diabetic collagen exhibited significantly more H_2_O_2_ compared to all other treatment groups, except non-diabetic cells treated with AGEs embedded in a diabetic matrix. Diabetic fibroblasts responded in a similar manner to non-diabetic cells; however, both AGE/RAGE signaling and matrix components had a significant impact on H_2_O_2_ production (Figure 8B; two-way ANOVA, Interaction *p* = 0.0092, Matrix *p* = 0.0097, and Treatment *p* = 0.0413). Diabetic cells embedded in a diabetic matrix and treated with AGE+EPAC had significantly more H_2_O_2_ than all other diabetic treatment groups in non-diabetic and diabetic matrices.

H_2_O_2_ concentration in Rap1a KO and RKO cardiac was not changed by treatment with exogenous AGEs, EPAC, or matrix components. Rap1a KO fibroblasts’ H_2_O_2_ concentration was not significantly altered by matrix components, exogenous AGEs, or EPAC treatment (Figure 8C; two-way ANOVA, Interaction *p* = 0.6933, Matrix *p* = 0.6476, and Treatment *p* = 0.6762). Likewise, H_2_O_2_ levels in non-diabetic RKO were not significantly impacted by exogenous AGEs, EPAC, or matrices (Figure 8D; two-way ANOVA, Interaction *p* = 0.9650, Matrix *p* = 0.9315, and Treatment *p* = 0.9908). Lastly, diabetic RKO fibroblasts’ regulation of H_2_O_2_ levels was not affected by pharmacological treatments or source of matrix (Figure 8E; two-way ANOVA, Interaction *p* = 0.8877, Matrix *p* = 0.8317, and Treatment *p* = 0.6032).

## 4. Discussion

The aim of this study was to examine the affect Rap1a has on mediating AGE/RAGE-induced cardiac fibroblast contraction, and the impact diabetic ECM has on facilitating this effect. We hypothesized that Rap1a contributes to the AGE/RAGE signaling cascade to induce cardiac fibroblast collagen gel contraction under diabetic conditions. The results from this study showed Rap1a crosses the AGE/RAGE cascade to contribute to cardiac fibroblast contraction. Furthermore, increased myofibroblast population, measured by elevated α-SMA expression, correlated with increased SOD-1 and SOD-2 expression, which could indicate a possible relationship between these factors to contribute to increased matrix contraction. Lastly, the results suggested that diabetic collagen elicited a differential response in cardiac fibroblasts as compared to non-diabetic collagen.

Previous studies by our lab demonstrated elevated AGEs within diabetic collagen mediated an increase in cardiac fibroblast contraction through increased RAGE signaling [7]. While the results presented in this study confirmed our previous findings, we found new evidence that indicates Rap1a is involved in regulating the amount of fibroblast contraction induced by AGE/RAGE signaling. EPAC treated non-diabetic and diabetic fibroblasts exhibited increased matrix contraction, which was demonstrated to be significant when EPAC treatment was combined with the addition of exogenous AGEs, as well as exposure to the endogenous AGEs found in the diabetic matrix. These results coincided with prior published studies that indicated Rap1a can modify signaling proteins linked to both the AGE/RAGE cascade and matrix contraction [5,30,62,63]. Specifically, Olmedo et al. 2013 conducted a study showing EPAC treatment of cardiac fibroblasts in a collagen matrix induced cell contraction [63]. The increase in matrix contraction could be a result of increased myofibroblast differentiation [6]. It has been well documented that AGE activation of RAGE can cause increased α-SMA expression, which serves as an indicator for myofibroblasts [6,7,26,29]. Our findings demonstrated a similar result in which α-SMA expression was increased as a result of increased AGE/RAGE signaling via exogenous AGE treatment and endogenous AGE exposure within the diabetic collagen. In addition, Rap1a contributed to increased α-SMA expression, which was supported by the results demonstrating there were no changes in α-SMA expression in Rap1a KO cells. Interestingly, α-SMA expression was higher in non-diabetic and diabetic fibroblasts embedded in a non-diabetic matrix. This finding could be due to either distinct resident populations of αSMA positive myofibroblasts within the isolated cardiac cells or timing of α-SMA protein expression to influence matrix contraction. α-SMA is considered to be a commonly measured indicator of myofibroblasts; however, expression of fibroblast activation protein (FAP) can also be used as a marker for fibroblast differentiation [24,64]. Studies have shown that expression of α-SMA and FAP do not tend to overlap in myofibroblast populations denoting separate populations of myofibroblasts [24,64,65]. While this could be a reason for the higher levels of α-SMA in non-diabetic matrices, a more plausible explanation is the timing of α-SMA protein expression and matrix contraction. Fibroblast-induced matrix contraction usually occurs within the first 24–48 h, marking a critical time for increased α-SMA expression [7,66]. After matrix contraction, myofibroblasts may transition back to a fibroblast phenotype or shift protein expression towards another downstream event, such as oxidative stress [21,67,68]. Additional studies will need to be conducted to examine the timing between increased α-SMA expression and matrix contraction in cardiac fibroblasts.

While Rap1a contributed to AGE/RAGE-mediated α-SMA matrix contraction, evidence also exists demonstrating that Rap1a may be able to impact cell mediated matrix contraction, possibly independent of AGE/RAGE signaling. This idea arose by Rap1a KO fibroblast displaying significantly more matrix contraction than non-diabetic and diabetic cells. Furthermore, α-SMA expression was lower in Rap1a KO cells and did not change when exposed to AGEs. Combining these results suggests that Rap1a may play more of a regulatory role regarding AGE/RAGE-mediated cellular contraction and can do so by altering additional signaling pathways, such as the RhoA/ROCK pathway [41,69,70,71]. Studies have shown that inhibition of Rap1b, which shares 95% homology with Rap1a, led to increased RhoA activity leading to phosphorylation of myosin regulatory light chain to increase cellular contraction in vascular smooth muscle cells [41,69,71,72]. In addition, Zieba et al. 2011 demonstrated that EPAC-treated gastric smooth muscle cells exhibited reduced RhoA/ROCK activity and decreased contraction of the cell, indicating a possible role for Rap1a within this pathway [71]. While Rap1a may influence RhoA/ROCK pathway independently of AGE/RAGE, studies that have shown AGE/RAGE signaling also utilizes the RhoA/ROCK pathway to modify cellular matrix contraction. For instance, AGE treatment to both human umbilical vein endothelial cells and microvascular endothelial cells caused an increase in RhoA/ROCK activity, which resulted in increased matrix contraction. Based on these findings, there may be some interplay between Rap1a and the AGE/RAGE cascade to regulate the RhoA/ROCK pathway mediated matrix contraction. Our results, in combination with the literature, indicated this as a possible mechanism. Further studies are needed to determine whether Rap1a and AGE/RAGE signaling utilize the Rho/ROCK cascade to modify cardiac fibroblast contraction.

While activation of RAGE signaling can induce changes in cardiac fibroblasts, a lack of RAGE, combined with cues from the ECM, can also exert an effect on fibroblasts. Fibroblasts that lacked RAGE (RKO) exhibited more matrix contraction when compared to fibroblasts expressing RAGE. These findings implicated RAGE activation as a key regulatory mechanism in determining the amount of collagen gel contraction in cardiac fibroblast. A similar pattern was noted with AGE/RAGE-mediated cardiac fibroblast migration, where very low and high levels of RAGE signaling resulted in an increase cardiac fibroblast migration [8]. In addition, Bierhaus et al. 2005 raised the idea of other mechanisms in RKO cells to compensate for the lack of RAGE [73]. The presence of an alternate mechanism may possibly explain increased contraction in RKO cells to regulate a specific signaling pathway, such as the RhoA/ROCK pathway [74,75]. The lack of the ability for AGE/RAGE signaling to regulate the RhoA/ROCK pathway may have also led to the elevated levels of α-SMA expression in RKO fibroblasts [76]. Tsapara et al. 2010 showed increased RhoA activity via TGF-β signaling led to increased α-SMA expression in retinal pigment epithelial cells [76]. Furthermore, studies have also shown RAGE signaling can interact with TGF-β signaling to influence fibroblast contraction and ECM remodeling, which are key functional characteristics of myofibroblasts [77]. TGF-β can also act independently of RAGE signaling to increase α-SMA expression in fibroblasts, which has been demonstrated to exacerbate fibrosis [78,79]. These studies highlighted another possible mechanism used by AGE/RAGE signaling to induce differing levels of cardiac fibroblast collagen gel contraction driven by myofibroblast differentiation.

AGE/RAGE signaling in combination with diabetic ECM induced an increase in oxidative stress indicators in cardiac fibroblasts. ROS production within cells can be upregulated when exposed to specific factors, such as high glucose levels or AGEs [5,11]. Increased ROS production, as well as increased RAGE signaling triggered by the presence of AGEs, can result in increased SOD expression [5,12,20]. SODs convert superoxides into hydrogen peroxide (H_2_O_2_), which can be further reduced by peroxidases to rid the body of reactive oxygen stressors [21,80]. Our results showed non-diabetic and diabetic fibroblasts exposed to EPAC, exogenous AGEs, and higher levels of endogenous AGEs found in diabetic collagen had significantly more expression of SOD-1, SOD-2, and therefore H_2_O_2_ levels [7,8]. These findings indicated AGEs and Rap1a mediated RAGE-induced SOD expression and H_2_O_2_ production in cardiac fibroblasts embedded in a 3D collagen matrix. These results aligned with data presented in the literature, where AGE treatment increased in RAGE associated proteins, such as PKC-ζ and NADPH oxidase, resulting in increased superoxide production and compensatory SOD expression [5,12,20,30].

An examination of expression of SODs in Rap1a KO fibroblasts suggested Rap1a was essential for AGE/RAGE-mediated increased SODs expression. Treatment of Rap1a KO fibroblasts with exogenous AGEs failed to produce an increase in SOD-1 expression; however, endogenous AGEs in the diabetic collagen led to more SOD-1 expression when compared to non-diabetic collagen. These results may indicate that the combined presence of AGEs and ECM induced SOD-1 expression in Rap1a KO cells. Adhesion of cells to the ECM components have also been shown to induce changes in ROS production and, in turn, SOD expression [39,81]. For instance, the binding of mouse embryonic fibroblasts to fibronectin via integrins α5β1 and ανβ3 induced an increase in ROS production [81]. While SOD-1 expression in Rap1a KO fibroblasts could be impacted by endogenous AGEs and ECM components, it appeared that SOD-2 expression could be regulated by a different mechanism in the absence of Rap1a. Our results showed SOD-2 expression was higher in Rap1a KO cells, but SOD-2 expression was not impacted by exogenous or endogenous AGEs. Rap1a could influence SOD-2 expression by mediating the activity of the NADPH oxidase complex [45,47,48,62]. Studies have demonstrated Rap1a phosphorylates p22^PHOX^_,_ a subunit of NADPH membrane complex, and can influence the assembly of the full NADPH complex by interacting with the p47^PHOX^ subunit to regulate ROS production [45,47,48,62]. Additionally, increased NADPH oxidase activity has been correlated to changes in SOD expression [82,83,84]. These observations may also explain our findings of higher levels of H_2_O_2_ in Rap1a KO cardiac fibroblasts. Based on this information, it appeared that Rap1a was essential for endogenous AGEs within the diabetic ECM to elicit a RAGE mediated increase in SOD expression.

Furthermore, our data also indicated the lack of RAGE did not prevent oxidative stress in cardiac fibroblasts. Our findings showed RKO fibroblasts exhibited significantly more H_2_O_2_ than fibroblasts with RAGE. While we did not directly examine the possible mechanism contributing to these results, we can postulate that the low level of SOD-1 expression in RKO fibroblasts caused a reduction in peroxidase activity, which reduced H_2_O_2_. Studies have shown a correlation between SOD-1 expression and peroxidase activity, such as glutathione peroxidase, in which low expression of SOD-1 correlated to low peroxidase activity [15,85,86,87]. Therefore, RKO fibroblasts may have increased H_2_O_2_ levels as a result of low peroxidase activity. Future studies will need to be conducted in order to determine whether low levels of SOD-1 expression were a contributing cause to higher H_2_O_2_ levels in RKO cardiac fibroblasts.

The composition and mechanical properties of isolated diabetic collagen affected cardiac fibroblast protein expression and function. The composition of collagen can differ based on the in vivo environment, where collagen isolated from diabetic cardiac tissue had a higher percentage of type III collagen [88]. Furthermore, the mechanical properties can also differ due to crosslinking either during collagen synthesis by lysyl oxidase or by glycation products, such as AGEs, in mature collagen [89,90,91]. Studies have shown that the composition of the ECM, along with its physical properties, can elicit different responses in cell protein expression and function. In our study, we saw that cardiac fibroblasts embedded in a diabetic matrix exhibited a different level of protein expression compared to cells in a non-diabetic matrix. These results could be due to fibroblasts interacting with the diabetic matrix via integrins to influence fibroblast adhesion, induction of specific signaling cascades, and alterations in cellular function [36,81,92,93]. For instance, the binding of fibroblasts to ECM by integrin subunit α5 can trigger mitochondrial release of ROS to induce signaling cascades, such as TGF-β, to promote expression of SODs and α-SMA [78,92,94]. This finding was further supported by data that indicated increased ROS production in cardiac fibroblasts correlated with increased collagen synthesis, a functional characteristic of α-SMA positive myofibroblasts [95]. In our study, the composition and mechanical properties of the diabetic collagen could have affected the expression and binding of specific integrins in the cardiac fibroblasts, which could have further propagated the affects induced by AGE/RAGE signaling in cardiac fibroblasts with RAGE, and contributed to the elevated levels of α-SMA and SOD-2 expression in RKO fibroblasts [81,88,96,97]. It appeared that diabetic collagen only elicited a response in cells that expressed Rap1a, as cardiac fibroblasts lacking Rap1a did not show differential responses between non-diabetic and diabetic collagens; thus, suggesting Rap1a played a crucial role in mediating signals from the extracellular diabetic collagen into intracellular responses. While Rap1a mediated these responses using the AGE/RAGE cascade, there may be other signaling proteins involved which have yet to be identified.

The goal of this study was to determine the affect Rap1a has on facilitating AGE/RAGE-induced cardiac fibroblast collagen gel contraction, as well as the impact diabetic collagen has on enabling this effect. The results from this study confirmed our hypothesis that Rap1a contributed to the AGE/RAGE signaling cascade to induce cardiac fibroblast collagen gel contraction under diabetic conditions. We determined that Rap1a was essential in mediating the response of cardiac fibroblasts to AGEs within the diabetic collagen. Elevated AGE/RAGE signaling induced increased α-SMA expression and oxidative stress, which resulted in increased fibroblast collagen gel contraction. Furthermore, we showed that knocking out either RAGE or Rap1a does not completely ablate these responses. This finding was most likely due to unidentified compensatory signaling cascades. If left unchecked AGE/RAGE signaling can lead to oxidative stress and myofibroblast differentiation, both key factors contributing to heart disease and eventual failure [5,6,15]. In order to identify and develop a potential therapeutic target to reduce the risk of cardiovascular disease in diabetics, we need to better understand the cellular mechanisms contributing to this risk. Based on the findings from this study, reduction, not ablation, of Rap1a could be a potential therapeutic target to reduce the level of AGE/RAGE signaling in diabetic conditions, and possibly reduce the risk for developing cardiovascular disease.

## Figures and Tables

**Table 1:** Physiological measurements of mice utilized in this study. The heart weight, body weight, and non-fasting blood glucose levels were measured before isolation of cardiac fibroblasts. The data presented consist of the mean ± SEM, and *n* value indicates number of mice used where 2–3 mouse hearts were used per cardiac fibroblast isolation. Statistical analysis consisted of one-way ANOVA followed by a Dunnett’s post hoc compared to non-diabetic mice (* *p* < 0.05 and **** *p* < 0.0001).

	Heart Weight	Blood Glucose (mg/dL)
	Body Weight	
Non-Diabetic (*n* = 25)	0.0038 ± 7.9128 × 10^−5^	190.6 ± 4.323
Diabetic (*n* = 18)	0.0017 ± 6.269 × 10^−5^ ****	546.1 ± 24.07 ****
Rap1a KO (*n* = 22)	0.0041 ± 11.18 × 10^−5^ *	180.0 ± 8.334
Non-Diabetic RKO (*n* = 20)	0.0037 ± 6.256 × 10^−5^	204.2 ± 4.454
Diabetic RKO (*n* = 13)	0.0022 ± 3.534 × 10^−5^ ****	456.4 ± 29.24 ****

## Data Availability

All relevant data are included within the manuscript. Supplemental and original blot images are available: https://doi.org/10.6084/m9.figshare.14150306 (accessed on 21 May 2021).

## Supplementary Materials

The following are available online at https://www.mdpi.com/article/10.3390/cells10061286/s1, Figure S1: 3D non-diabetic collagen matrix induced changes in cell morphology in cardiac fibroblasts independently of RAGE, Figure S2: Diabetic collagen displayed reduced light transmission and matrix compaction compared to non-diabetic collagen, and Figure S3: Cardiac fibroblasts embedded in non-diabetic and/or diabetic 3D collagen matrix rearranged to form a contractile ring connected by a cellular network.
