# Supplementary material for: Rap1a Regulates Cardiac Fibroblast Contraction of 3D Diabetic Collagen Matrices by Increased Activation of the AGE/RAGE Cascade
Source: Cells. 2021 May 22;10(6):1286. doi: 10.3390/cells10061286 (PMC8224555; doi:10.3390/cells10061286)
Supplement: Supplementary file 1 [file cells-10-01286-s001.zip › cells-1169548-supplementary.pdf]

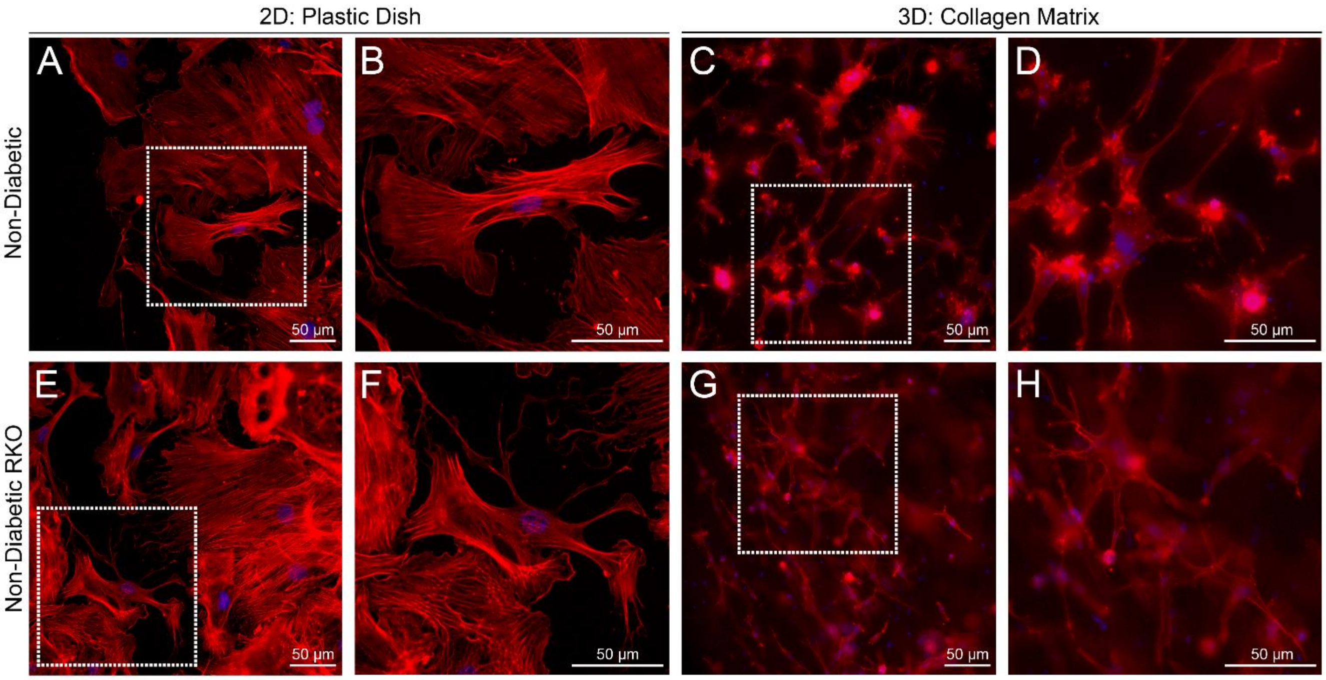

Figure S1. 3D non-diabetic collagen matrix induced changes in cell morphology in cardiac fibroblasts independently of RAGE. (**A**–**D**) Non-diabetic and (**E**–**H**) non-diabetic RKO fibroblasts were isolated from mouse hearts. Cardiac fibroblasts were either cultured on (**A**, **B**, **E**, **F**) plastic cell culture dish or were (**C**, **D**, **G**, **H**) embedded in a non-diabetic collagen matrix. After 24, cells were fixed and labeled with Phalloidin (red; actin) and DAPI (blue; nucleus). Images A, C, E, and G were taken at 20× with a scale bar of 50 µm. The white dotted box depicts the section of the image that was enlarged (B, D, F, and H; scale bar = 50 µm).

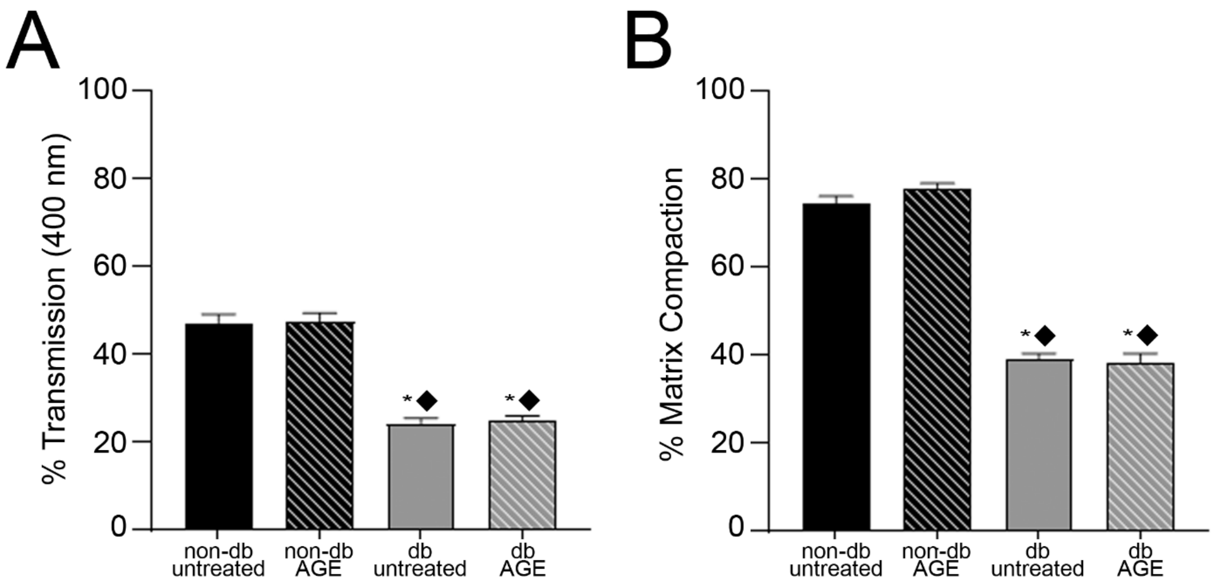

Figure S2. Diabetic collagen displayed reduced light transmission and matrix compaction compared to non-diabetic collagen. Collagen was isolated from tails collected from non-diabetic (non-db) and diabetic (db) mice (~30 tails). Isolated collagen was either combined with or without exogenous AGEs (0.5 mg/mL) and allow to polymerize for 1 hour before experimental assessment. (**A**) Percent transmission at 400 nm and (**B**) percent matrix compaction was assessed in untreated (solid bars) and exogenous AGE (striped bars) treated non-diabetic (non-db) and diabetic (db) matrices. Mean ± SEM depicted in graphs with n = 22 replicates. Statistical analysis consisted of a one-way ANOVA followed by a Fisher's Least Significant Difference post hoc ($p < 0.05$; * vs untreated non-db matrix and ◆ vs AGE non-db matrix).

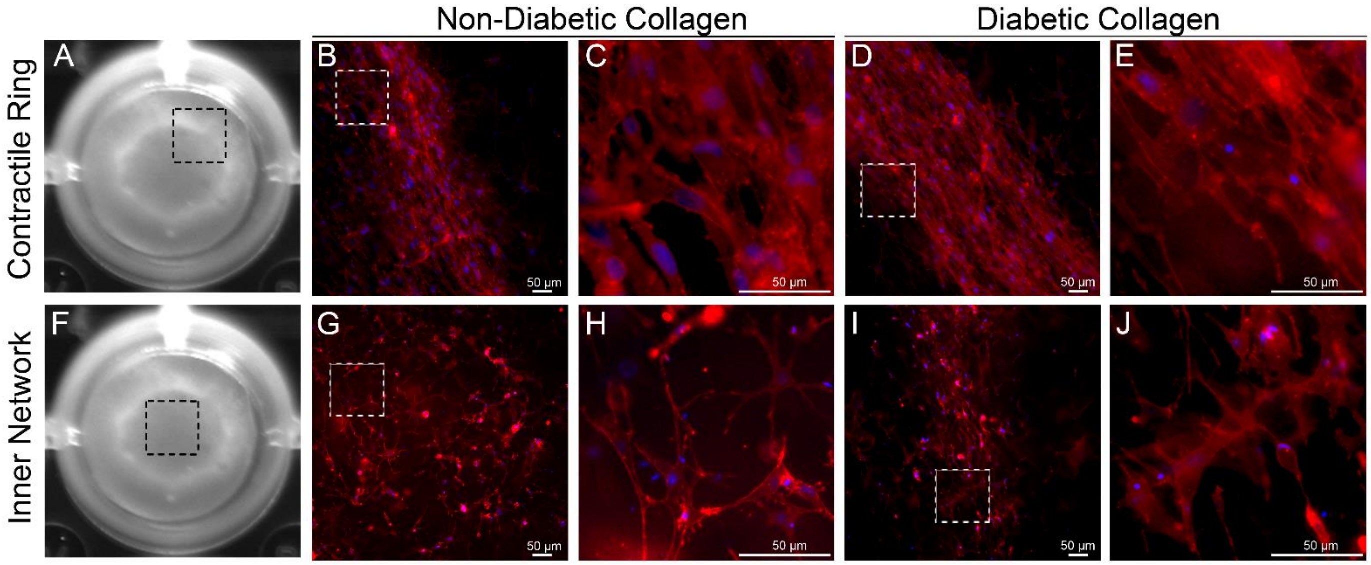

Figure S3. Cardiac fibroblasts embedded in non-diabetic and/or diabetic 3D collagen matrix rearranged to form a contractile ring connected by a cellular network. Non-diabetic fibroblasts were isolated from

mice hearts and embedded in either a non-diabetic or diabetic matrix for 24 hrs. Cells/matrix were fixed and labeled with Phalloidin (red; actin) and DAPI (blue; nucleus). (**A–E**) Images depicting the edge of the contractile ring formed by fibroblasts in non-diabetic and diabetic matrices. (**F–J**) Images of the inner section of the contractile ring. Images were taken at 10X with a scale bar = 50 μM (**A**, **C**, **E**, **G**) and the white dotted box depicts the section of the image that was enlarged (**B**, **D**, **F**, **H**; scale bar = 50 μM). (**A** and **F**) Representative matrix images denote the corresponding area that is presented in the following immunofluorescence images.
